# Commentary: Immune-related intestinal pseudo-obstruction caused by immune checkpoint inhibitors: case report

**DOI:** 10.3389/fonc.2025.1543608

**Published:** 2025-05-23

**Authors:** Jie-Qiang Xia, Jian Fan, Guang-Qing Shi, Hao Sun

**Affiliations:** ^1^ Department of Neurology, The First People’s Hospital of Shuangliu District (West China Airport Hospital of Sichuan University), Chengdu, Sichuan, China; ^2^ Department of Respiratory and Critical Care Medicine, The General Hospital of Western Theater Command, Chengdu, Sichuan, China

**Keywords:** immune checkpoint inhibitor, paraneoplastic neurologic syndromes, gastrointestinal pseudo-obstruction, PNSs, PNS-Care Score

## 1 Introduction

Recent years have shown that immune checkpoint inhibitors (ICIs) increased survival and long-term remissions, even in patients with extensive metastatic cancer (1, 2). However, the resulting increase in T cell effector function often results in immune-related adverse events (irAEs), some affecting the nervous system (nirAEs) (3, 4), a subset of which constitute classical paraneoplastic neurological syndromes (PNSs) (3). High-risk neurological phenotypes of PNSs include encephalomyelitis, limbic encephalitis, rapidly progressive cerebellar syndrome, opsoclonus myoclonus syndrome, subacute sensory neuronopathy, gastrointestinal pseudo-obstruction (GIPO, enteric neuropathy), and Lambert-Eaton myasthenic syndrome (1). We have previously reported two cases of patients with small-cell lung cancer (SCLC) who developed PNSs induced by ICIs (5), which prompted our focus on studies related to irAEs of ICIs, particularly nirAEs. In this context, we were greatly interested in the recent publication by Qian et al., titled “Immune-related intestinal pseudo-obstruction caused by immune checkpoint inhibitors: case report,” which describes a patient with hepatocellular carcinoma (HCC) developed intestinal pseudo-obstruction after treatment with pembrolizumab (6). We appreciate the authors for sharing their valuable clinical insights and experiences. We would like to propose adding some considerations regarding their paper, which we hope to discuss with the authors.

## 2 Commentary and discussion

The authors regard this patient’s situation as immune therapy-related intestinal obstruction. However, we propose an alternative interpretation of this patient’s intestinal obstruction, which was a PNS induced by ICIs therapy.

Distinguishing between irAEs and PNSs presents significant challenges, and most clinical trial reports do not clearly define whether some of the observed nirAEs are in fact PNSs (3). The 2021 expert panel recommends that the diagnosis of PNS be established using the PNS-Care Score, which considers the clinical phenotype, the presence or absence of neuronal antibodies, and the presence or absence of cancer, while systematically excluding other alternative etiologies (1). In patients receiving ICIs when neurological symptoms occur, it is essential to assess both the presence of neuronal antibodies and the PNS-Care Score to ascertain whether the symptoms are attributable to an irAE or a PNS (1). Based on the score, this case fulfilled the diagnostic criteria for probable PNS. There were no abnormalities in the intestinal tract prior to ICI administration, and the PNS manifested after six cycles of ICI therapy, with no other causes of intestinal obstruction identified. Notably, according to the 2004 diagnostic criteria for PNS, when a cancer patient exhibits symptoms of a nirAE after receiving ICIs, these symptoms fulfill the criteria for PNS if they are identical to classical PNS symptoms, regardless of the presence of neuronal autoantibodies (3, 7). The 2021 criteria refined diagnostic accuracy through a stringent scoring system, but at the cost of sensitivity. In this case, we inferred that the PNS was induced by the ICI. Regrettably, the patient was not tested for neuronal antibodies, which could have provided further clarity on the diagnosis.

Enteric neuropathy, also known as paraneoplastic gastrointestinal paresis or chronic intestinal pseudo-obstruction, is a high-risk neurological phenotype of PNSs, usually associated with anti-Hu antibody (Hu-Ab) and SCLC (3). Reports have also indicated the presence of Hu-Ab in patients with HCC (8). HCC with paraneoplastic GIPO is a rare presentation. However, nirAEs such as encephalitis (9) and Guillain-Barré syndrome (10) have been reported in HCC patients after treatment with ICIs. The pathology of GIPO is that Hu-Ab causes irreversible dysfunction of the enteric plexus along with lymphoplasmacytic infiltration (11). There are limited accounts of GIPO potentially resulting from ICI-induced PNS along with positive anti-Hu antibodies. Kang et al. described a case of SCLC methylprednisolone for sintilimab-induced encephalitis that was started a few days before the onset of GIPO, cerebrospinal fluid Hu-Ab positive, and it is possible that GIPO improved because of early therapeutic intervention (12). In another report by Saitou et al., a case of SCLC with GIPO elicited by durvalumab and serum Hu-Ab was positive. We have recently reported a similar case (13). However, corticosteroid treatment did not improve the GIPO (11). Post-immune checkpoint inhibitor PNSs are usually unresponsive to corticosteroids and lead to severe neurological disability and high mortality (4). Anecdotal reports and small case series indicate that patients can have symptom improvements with intravenous immunoglobulin(IVIG) therapy (3). Neuronal antibodies have important implications for the early diagnosis of PNSs. The potential benefits of conducting neural antibody screening in cancer patients likely to experience PNSs before initiating ICIs merit further investigation (4).

In summary, it is important to recognize that GIPO is one of the high-risk phenotypes of PNS. From the viewpoint of neurologists, we propose an alternative diagnosis to enhance the understanding of ICI treatment-induced PNS. The PNS-Care Score may help differentiate irAEs from PNS. Furthermore, routine neuronal antibody testing is recommended to improve the diagnosis of PNSs occurring in the context of ICI therapy for all patients who develop neurologic irAEs that resemble high or intermediate risk PNS (1). Patients experiencing such toxicities should be evaluated by neurologists with expertise in neuroimmunology.
